# Polyphenol and Flavonoid Levels of Selected Morello and Amarelle Tart Cherry Cultivars (*Prunus cerasus* L.): Implications for Orchard Production and Consumer Selection

**DOI:** 10.3390/molecules31162881

**Published:** 2026-08-18

**Authors:** Justyna Maria Traore, Elżbieta Rozpara, Agnieszka Głowacka, Witold Danelski, Ewelina Hallmann, Alicja Ponder, Małgorzata Żebrowska-Krasuska, Klaudia Kopczyńska, Dominika Średnicka-Tober, Renata Kazimierczak

**Affiliations:** 1 Department of Functional and Organic Food, Institute of Human Nutrition Sciences, Warsaw University of Life Sciences, 02-776 Warsaw, Poland; justynatraore@gmail.com (J.M.T.); ewelina_hallmann@sggw.edu.pl (E.H.); alicja_ponder@sggw.edu.pl (A.P.); malgorzata_zebrowska@sggw.edu.pl (M.Ż.-K.); klaudia_kopczynska@sggw.edu.pl (K.K.); dominika_srednicka_tober@sggw.edu.pl (D.Ś.-T.); 2 Cultivar Testing, Nursery and Gene Bank Resources Department, The National Institute of Horticultural Research, 96-100 Skierniewice, Poland; elzbieta.rozpara@inhort.pl (E.R.); agnieszka.glowacka@inhort.pl (A.G.); witold.danelski@inhort.pl (W.D.); 3 Bioeconomy Research Institute, Agriculture Academy, Vytautas Magnus University, Donelaicio 58, 44248 Kaunas, Lithuania

**Keywords:** tart cherry cultivars, sour cherry, Morello Cherry, Amarelle Cherry, polyphenols, phenolic acids, flavonoids, anthocyanins

## Abstract

Tart cherries (*Prunus cerasus* L.) are valued for their high content of bioactive compounds, particularly polyphenols, flavonoids, and anthocyanins, which contribute to fruit quality and potential health benefits. However, data on phytochemical differences between Morello and Amarelle cultivars remain limited. In the present study, eleven tart cherry cultivars representing both groups were evaluated over two growing seasons (2024–2025) under identical orchard conditions. Dry matter, phenolic acids, flavonoids, and anthocyanins were quantified using HPLC methods, and the effects of cultivar, season, and their interaction were assessed by ANOVA. Significant differences were observed among cultivars and between seasons (*p* < 0.05). Kelleris 16 showed the greatest concentrations of total polyphenols and phenolic acids, whereas Łutówka was characterized by elevated levels of catechin, epigallocatechin, quercetin derivatives, and myricetin. Morello cultivars generally contained substantially higher flavonoid and anthocyanin concentrations than Amarelle cultivars. In contrast, selected Amarelle cultivars, particularly Szklanka Wielka, exhibited higher levels of chlorogenic and caffeic acids. Most polyphenolic compounds were more abundant in 2024 than in 2025, highlighting a strong seasonal effect, while anthocyanin levels remained comparatively stable in both seasons. Both genotype and growing season significantly influenced the phytochemical composition of tart cherries. Morello cultivars generally exhibited higher flavonoid and anthocyanin concentrations than Amarelle cultivars, whereas selected Amarelle cultivars contained higher levels of specific phenolic acids. These findings provide valuable information for cultivar selection and future studies on the nutritional and functional properties of tart cherry fruits.

## 1. Introduction

Tart cherries (*Prunus cerasus* L.) (sour cherries) represent an economically and nutritionally important fruit group cultivated globally for both fresh consumption and processing into juices, concentrates, preserves, and nutraceutical products. Their distinctive sensory characteristics—high acidity, intense pigmentation, and robust flavor profile—are coupled with a phytochemically rich matrix dominated by anthocyanins and other polyphenols that has attracted substantial interest in the context of human health and functional food development [1,2]. Among the diverse tart cherry germplasm, the Morello and Amarelle types constitute historically and commercially significant varietal groups that differ in fruit color, morphology, phenology, and organoleptic properties. Given their widespread cultivation in temperate regions and their use in both fresh and processed markets, accurate characterization of their nutritional and bioactive profiles is essential for informed orchard management and consumer product selection [3].

Extensive analytical studies have demonstrated that tart cherries are abundant in anthocyanins; hydroxycinnamic acids; flavanols; flavan-3-ols; proanthocyanidins; vitamin C; and organic acids, particularly malic acid [1,4,5]. Their anthocyanin profile is typically dominated by cyanidin-based glycosides, which contribute to the characteristic dark red pigmentation and the high antioxidant activity observed in vitro. Reported concentrations of total polyphenols typically range from several hundred mg gallic acid equivalents (GAE) per 100 g FW (fresh weight), whereas anthocyanin concentrations often reach tens of mg cyanidin-3-glucoside equivalents per 100 g FW, though these values vary substantially among cultivars and growing regions [4,5,6]. This rich phytochemical composition underlies the functional properties attributed to tart cherry products, highlighting the importance of cultivar-specific chemical profiling.

A recurring theme across compositional surveys is that genotype is one of the strongest determinants of phytochemical content, frequently surpassing the effects of environment and season [5,7]. Comparative analyses involving numerous *P. cerasus* cultivars have revealed wide ranges in total phenolic content, individual anthocyanins, soluble solids, titratable acidity, and sugar-acid ratios, each of which contributes to fruit quality, sensory experience, and processing behavior [5,7,8].

Morello-type cherries are typically characterized by darker pigmentation, higher juice coloration potential, and late ripening, while Amarelle-type cherries often present brighter red or light-tinted juice and distinct tree morphology. Some Amarelle cultivars exhibit notably high phenolic concentrations and antioxidant capacity, while certain Morello genotypes demonstrate elevated anthocyanin content and strong antiglycative or antioxidant activity in functional assays [3,9]. These cultivar-specific differences underscore the need for direct comparison of representative varieties to inform both agricultural and consumer-facing decisions.

Post-harvest handling significantly affects the retention of nutritional and bioactive compounds in tart cherries. Thermal treatments, prolonged storage, and suboptimal handling accelerate degradation of anthocyanins and other labile polyphenols, whereas rapid cooling, freezing, or mild processing methods improve phytochemical preservation [10,11]. From an orchard management perspective, optimal harvest timing and cold chain integrity are crucial for ensuring maximum bioactive retention. For processors, cultivar selection with inherently higher concentrations of stable pigments may provide technological advantages for products such as juices, concentrates, or dried cherries.

The compositional richness of tart cherries has spurred interest in their potential health effects. Human and animal studies report benefits related to inflammation, oxidative stress, exercise-induced muscle damage, and sleep regulation, although the magnitude and consistency of effects vary across trials [11,12,13,14]. Mechanistic work often implicates anthocyanin-mediated modulation of oxidative and inflammatory pathways. However, outcomes depend heavily on the phytochemical dose, product format (whole fruit, juice, concentrate, powder), and cultivar-specific composition. Thus, it is increasingly recognized that not all tart cherry products are equivalent to their health-relevant properties.

Recent genomic and germplasm analyses of *P. cerasus* have facilitated improved characterization of cultivars, enabling marker-assisted breeding strategies aimed at enhancing both agronomic performance and phytochemical quality [15,16]. The integration of compositional data with horticultural traits, such as yield, pest resistance, ripening time, and stress tolerance, provides growers with evidence-based tools for selecting cultivars suited to specific production and market needs. For consumers and food manufacturers, such information supports more accurate labelling, targeted functional claims, and informed product development.

Despite substantial progress in sour cherry research, there is still a lack of harmonized, side-by-side comparisons of Morello and Amarelle cultivars using standardized, high-resolution analytical techniques. Few studies directly link orchard management conditions to detailed nutritional and phytochemical compositions or to consumer-relevant attributes, such as color stability and soluble solids-to-acidity balance. The objective of the present study is therefore to quantify and compare the nutritional components and polyphenol profiles of selected Morello and Amarelle tart cherry cultivars, and to interpret these findings in the context of orchard production and informed consumer choice.

## 2. Results and Discussion

### 2.1. Dry Matter

Kelleris 16 (Morello-type) exhibited the highest average dry matter content across both experimental years (14.06 ± 0.36 g/100 g FW), followed by Sabina (13.33 ± 0.42 g/100 g FW) and Szklanka Wielka (Amarelle-type) (13.18 ± 0.43 g/100 g FW). The lowest mean levels of dry matter showed the following Amarelle cultivars: Galena (11.64 ± 0.37 g/100 g FW), Early Richmond (11.58 ± 0.37 g/100 g FW) and Richmorency (11.07 ± 0.20 g/100 g FW). The seasonal average across all cultivars was higher in 2025 by approximately 0.56 g/100 g FW.

The observed differences in dry matter content among cultivars indicate a strong genotypic effect on fruit composition. Kelleris 16 consistently exhibited the highest dry matter content, suggesting greater accumulation of soluble solids and structural compounds during fruit development. Previous studies have demonstrated that dry matter content in sour cherries is influenced not only by cultivar characteristics but also by environmental conditions, particularly temperature and water availability during fruit ripening [17,18]. The higher average dry matter content observed in 2025 may reflect seasonal differences in climatic conditions, potentially leading to increased concentrations of fruit constituents. From a food processing perspective, cultivars with higher dry matter content may offer advantages to processing industries, due to improved extraction yields and enhanced sensory properties of processed products.

### 2.2. Phenolic Compounds

Higher mean values of total polyphenols were observed in all cultivars during the season 2024 (approx. 100 mg/100 g FW) compared with the season 2025 (approx. 72 mg/100 g FW). Phenolic acid levels were about twice as high across all cultivars in 2024 (approx. 31 mg/100 g FW) compared with 2025 (approx. 15 mg/100 g FW). The highest overall average concentrations of polyphenols, as well as phenolic acids, were observed in Kelleris 16—a tart cherry cultivar reported to be the most susceptible to infections and frost in a different study [19].

**Figure 1:**
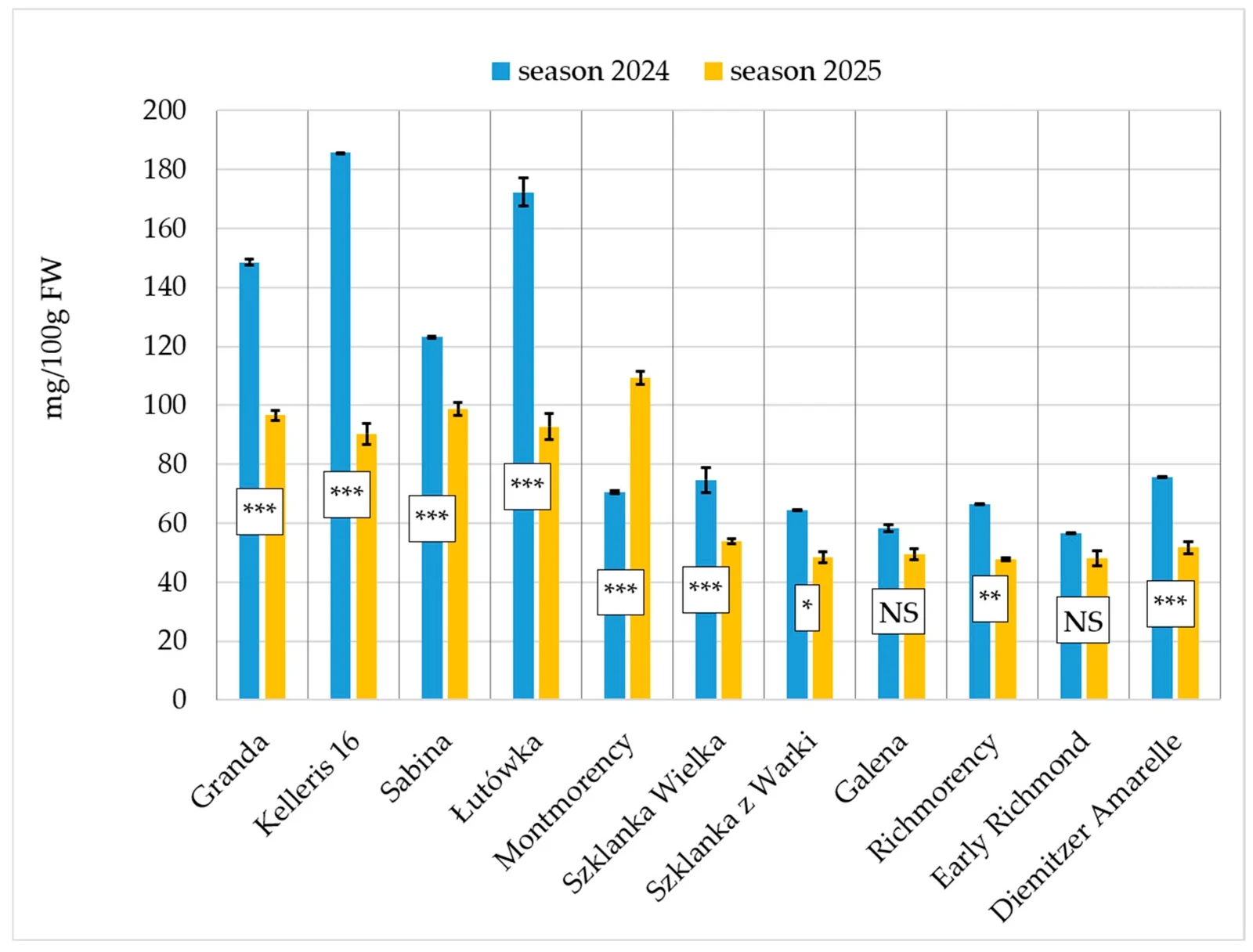
Mean values ± SE of total polyphenols content in different tart cherry cultivars over two experimental years (2024–2025); *p*-value < 0.0001 in the ANOVA test for the influence of cultivar, year and their interaction. Signs on the bars for each cultivar indicate significant differences between years (*p*-value (*) <0.05, (**) <0.01, (***) <0.001). NS—no significant differences.

**Figure 2:**
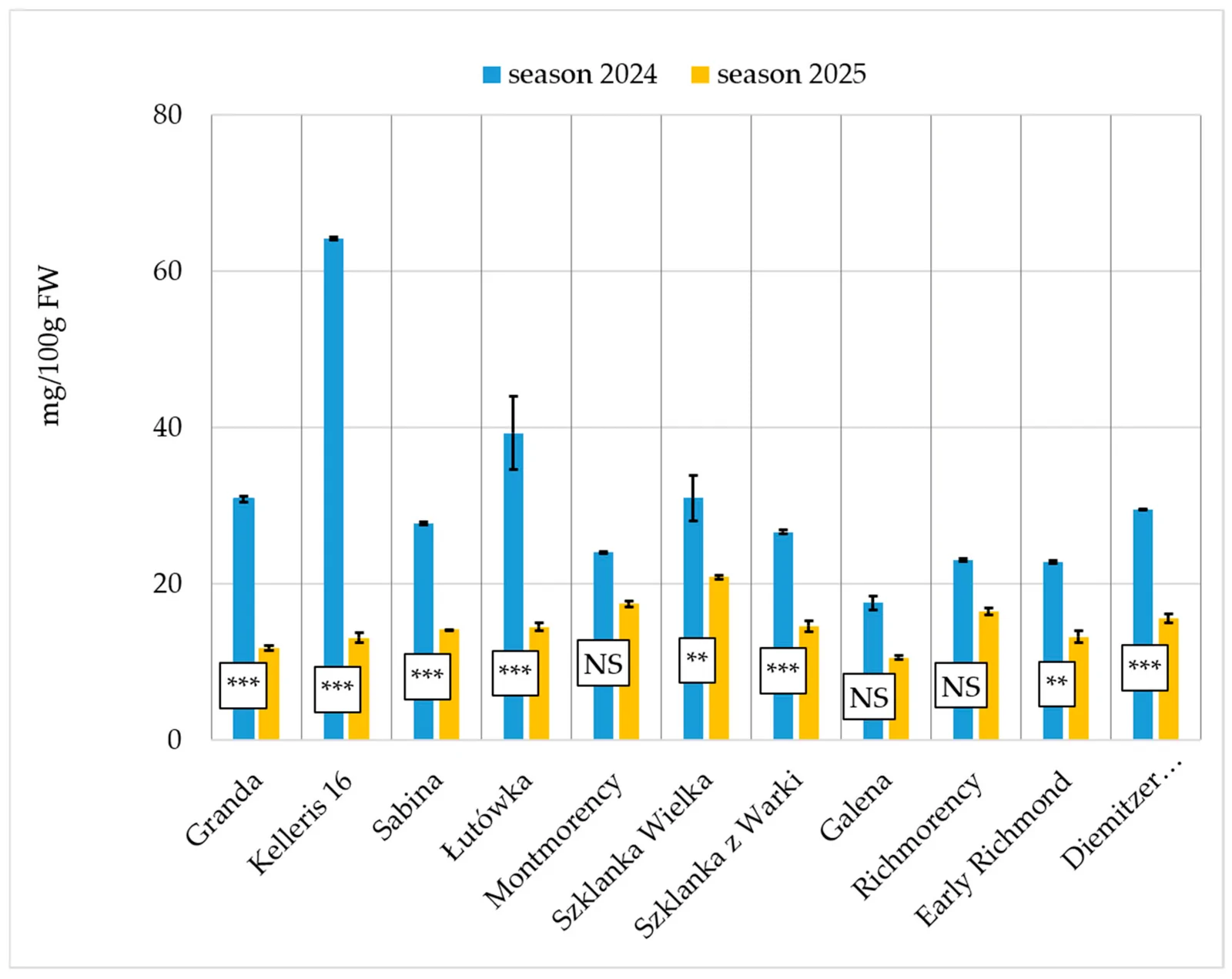
Mean values ± SE of total phenolic acid content in different tart cherry cultivars over two experimental years (2024–2025); *p*-value < 0.0001 in the ANOVA test for the influence of cultivar, year and their interaction. Signs on the bars for each cultivar indicate significant differences between years (*p*-value (**) <0.01, (***) <0.001). NS—not significant differences.

Higher concentrations of total polyphenols and phenolic acids were generally observed in 2024 than in 2025 (Figure 1 and Figure 2). These differences indicate a pronounced seasonal effect on phytochemical accumulation, although they cannot be attributed to a single climatic factor. Phenolic biosynthesis is regulated by a complex interaction between genotype and environmental conditions, including temperature, rainfall distribution, solar radiation, water availability during fruit development, fruit maturity at harvest, and plant responses to abiotic stress [20,21]. Variations in these factors between the two growing seasons may have influenced both the synthesis and accumulation of phenolic compounds, as well as possible dilution effects associated with differences in fruit growth and fruit water content [22,23].

Interestingly, Kelleris 16 exhibited the highest concentrations of both total polyphenols and phenolic acids despite its reported susceptibility to frost and pathogen infection [19]. This finding suggests that the elevated accumulation of phenolic compounds may reflect cultivar-specific regulation of the phenylpropanoid pathway rather than enhanced stress tolerance. Phenolic metabolites play an important role in antioxidant defense, pathogen inhibition, and cell wall reinforcement [24]. Accordingly, the high phenolic content observed in Kelleris 16 may represent a biochemical response associated with stress adaptation rather than an indicator of greater resistance. However, because stress tolerance was not directly evaluated in the present study, this relationship should be interpreted with caution and requires further investigation. Nevertheless, cultivars characterized by high phenolic content may represent valuable genetic material for breeding programs aimed at improving fruit quality. Further studies are needed to determine whether these phytochemical traits are associated with enhanced tolerance to environmental stress.

Furthermore, although the present study was not designed to quantify relationships between individual climatic variables and phytochemical composition, future studies integrating detailed meteorological data with correlation analyses would provide a better understanding of the environmental regulation of phenolic biosynthesis.

#### Individual Phenolic Acids

Individual phenolic acids showed considerable cultivar- and season-dependent variation (Tables S1 and S2 in the Supplementary Materials). Among hydroxybenzoic acids, gallic acid reached the highest concentrations in Łutówka, with mean values approximately three times higher than those recorded in Galena. The greatest seasonal differences were observed in Kelleris 16 (approximately 12 mg/100 g FW), Łutówka (8.9 mg/100 g FW), Granda (7.5 mg/100 g FW), and Sabina (7.2 mg/100 g FW), whereas Montmorency and Early Richmond were characterized by relatively comparable concentrations in both experimental seasons.

Among hydroxycinnamic acids, *p*-coumaric acid exhibited the greatest cultivar-dependent variation. Kelleris 16 contained the highest concentrations, reaching approximately 43.5 mg/100 g FW in 2024, which was about three times higher than the seasonal average. Most cultivars showed markedly lower concentrations in 2025, whereas Richmorency exhibited only a slightly lower levels, and recorded the highest *p*-coumaric acid concentration during the second growing season.

Ferulic acid concentrations varied less markedly than *p*-coumaric acid. The highest ferulic acid levels in 2024 were observed in Granda, Early Richmond, and Szklanka z Warki, whereas Kelleris 16 maintained similar concentrations in both seasons. In contrast, Sabina, Szklanka Wielka, and Diemitzer Amarelle exhibited increased ferulic acid concentrations in 2025, while the remaining cultivars showed relatively small seasonal differences, with mean values generally ranging between 1.1 and 1.5 mg/100 g FW.

Clear cultivar-related differences were also observed for chlorogenic acid. Szklanka Wielka consistently exhibited the highest chlorogenic acid concentrations, approximately three times higher than those of Early Richmond, whereas Sabina showed the highest values among Morello cultivars. Seven of the eleven cultivars exhibited lower chlorogenic acid concentrations in 2025, with the greatest between-season differences recorded in Richmorency and Montmorency. In contrast, concentrations in fruits of Szklanka z Warki remained almost unchanged between seasons.

Caffeic acid clearly differentiated the two tart cherry groups. Amarelle cultivars generally contained higher caffeic acid concentrations than Morello cultivars, with Szklanka Wielka consistently exhibiting the highest values and Sabina the lowest. Most Amarelle cultivars showed slightly higher contents in 2025, particularly Montmorency, Szklanka z Warki, and Diemitzer Amarelle, whereas seasonal differences in the remaining cultivars were relatively small.

Among the identified phenolic acids, *trans*-cinnamic acid occurred at the lowest concentrations. Montmorency exhibited the highest *trans*-cinnamic acid level in 2024 (0.68 mg/100 g FW), which was approximately 8.5-fold lower in 2025. Richmorency consistently showed the lowest concentrations in both seasons, whereas seasonal variation in the remaining cultivars was generally limited.

The present study demonstrated pronounced cultivar- and season-dependent variation in the phenolic acid composition of tart cherry fruits. The overall reduction in the concentrations of most phenolic acids in 2025 indicates that environmental conditions substantially influenced phenolic acid biosynthesis, although the magnitude of these changes differed among cultivars. Similar seasonal variability has previously been reported for sour cherries, stone fruits, and other *Prunus* species, where fluctuations in temperature, precipitation, fruit maturity, and other environmental factors affected the accumulation of phenolic compounds [20,25,26].

Among the analyzed cultivars, Kelleris 16, Łutówka, Sabina, and Szklanka Wielka were distinguished by characteristic phenolic acid profiles. Kelleris 16 exhibited exceptionally high *p*-coumaric acid concentrations, whereas Łutówka accumulated the highest amounts of gallic acid. Szklanka Wielka consistently contained the highest concentrations of chlorogenic and caffeic acids, while Montmorency was characterized by elevated *trans*-cinnamic acid levels. The persistence of these cultivar-specific patterns across both growing seasons suggests that genotype is a major determinant of phenolic acid composition, although environmental conditions clearly modulated the accumulation of individual compounds.

The observed variability among individual phenolic acids also reflects differences in their metabolic regulation. *P*-coumaric acid is a key intermediate of the phenylpropanoid pathway and a precursor of flavonoids, lignin, and anthocyanins [27,28]. Therefore, the exceptionally high concentrations observed in Kelleris 16 may partially explain the elevated total polyphenol content recorded in this cultivar. In contrast, chlorogenic and caffeic acids accumulated predominantly in Amarelle cultivars, particularly Szklanka Wielka, indicating cultivar-specific regulation of hydroxycinnamic acid biosynthesis. Similar genotype-dependent differences in phenolic acid accumulation have been reported previously in sour cherries and related *Prunus* species [26,29,30,31].

Although gallic, ferulic, chlorogenic, caffeic, and *trans*-cinnamic acids are recognized as important contributors to the antioxidant properties of fruits and participate in plant defense and stress-response mechanisms [32,33,34,35,36], the present study focused exclusively on their quantitative composition. Therefore, the observed differences should primarily be interpreted as evidence of cultivar- and season-dependent variation in phytochemical profiles rather than direct indicators of biological activity. Nevertheless, the characteristic phenolic acid composition identified for several cultivars may provide useful information for future studies investigating their nutritional quality and functional potential.

Overall, the results indicate that both genetic background and growing season contributed to the phenolic acid profile of tart cherry fruits, with genotype determining the characteristic composition of individual cultivars and environmental conditions modulating the accumulation of specific compounds. These findings are consistent with previous reports describing the combined effects of genotype and environmental factors on phenolic metabolism in cherry fruits [17,20,25,26,30,37,38].

### 2.3. Flavonoids

**Figure 3:**
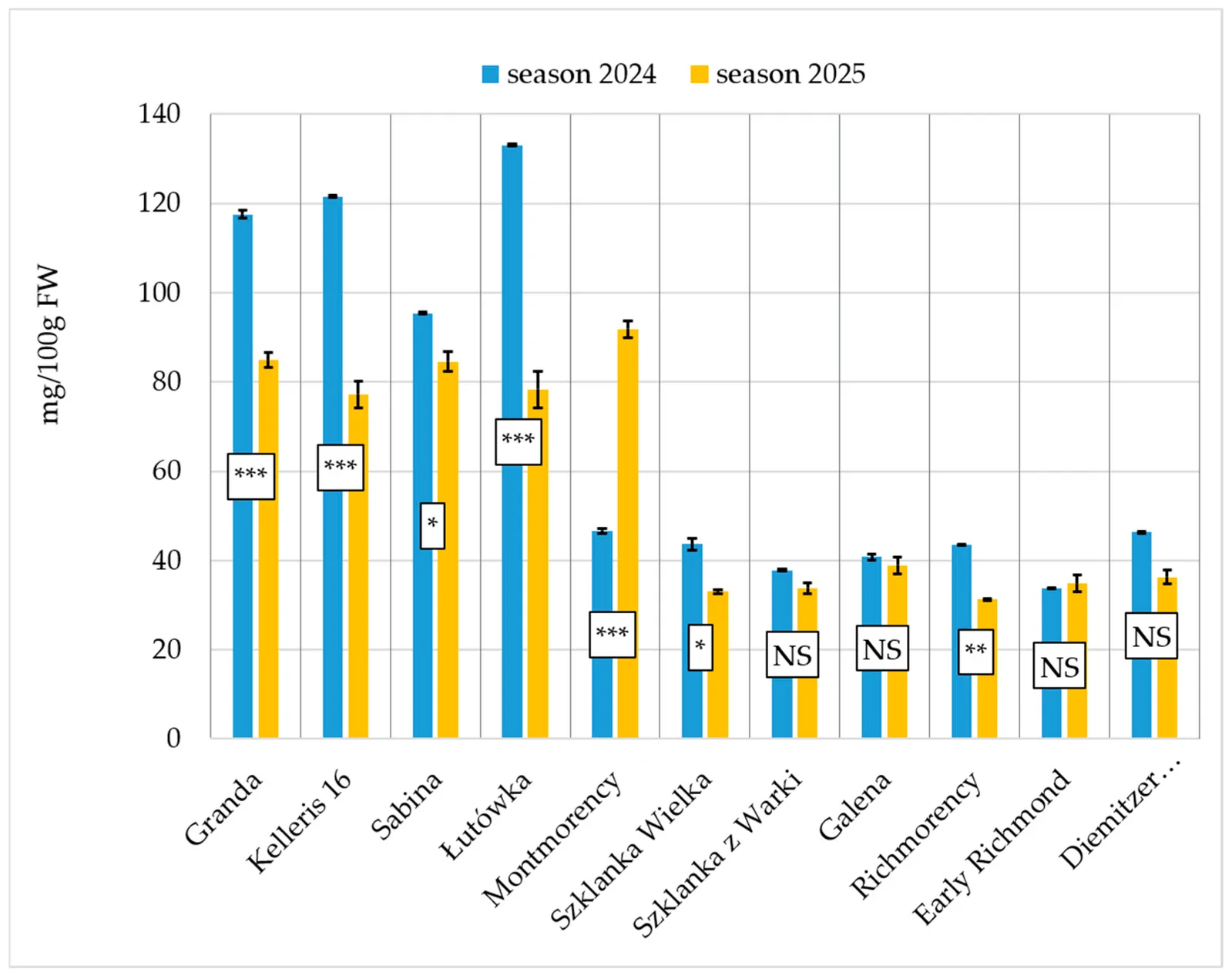
Mean values ± SE of total flavonoid (including anthocyanins) content in different tart cherry cultivars over two experimental years (2024–2025); *p*-value < 0.0001 in the ANOVA test for the influence of cultivar, year and their interaction. Signs on the bars for each cultivar indicate significant differences between years (*p*-value (*) <0.05, (**) <0.01, (***) <0.001). NS—not significant differences.

The concentration of flavonoids (sum) in most cultivars was higher in the 2024 season than in 2025 (Figure 3). Morello cherries (Granda, Kelleris 16, Sabina, and Łutówka) contained, on average, between approximately 95 and 133 mg/100 g FW of total flavonoids, whereas Amarelle cherries (Szklanka Wielka, Szklanka z Warki, Galena, Richmorency, Early Richmond, and Diemitzer Amarelle) contained between approximately 33 and 47 mg/100 g FW. The highest flavonoid concentration was recorded for Łutówka in 2024, while the lowest value was observed for Richmorency in the same season. The overall lower flavonoid concentration in 2025 corresponded with the lower total polyphenol content, indicating that seasonal conditions affected the accumulation of bioactive compounds in sour cherries.

The substantially higher flavonoid concentrations observed in Morello cultivars compared with Amarelle cultivars indicate clear genetic differences in secondary metabolite accumulation. Similar differences between cultivars have been widely observed in sour cherry collections [39,40]. Since flavonoids contribute significantly to antioxidant activity and fruit coloration, cultivars such as Łutówka, Kelleris 16, and Sabina may offer enhanced nutritional value and greater consumer appeal for those seeking functional foods.

#### Individual Flavonoids

Individual flavonoids showed considerable cultivar-dependent variation (Tables S3 and S4 in the Supplementary Materials). Epigallocatechin concentrations clearly differentiated tart cherry groups. Morello cultivars generally contained higher epigallocatechin concentrations than Amarelle cultivars, with Łutówka consistently exhibiting the highest levels, approximately six times higher than the lowest values recorded for Early Richmond. Although slight seasonal differences were observed among individual cultivars, the overall epigallocatechin concentration remained relatively stable between the two growing seasons, indicating lower seasonal variability than for most other flavonoids.

Catechin concentrations also varied among cultivars and between growing seasons. Łutówka consistently exhibited the highest catechin content, followed by Kelleris 16, whereas the lowest concentrations were recorded in Richmorency and Early Richmond. Most cultivars showed lower catechin concentrations in 2025, although seasonal variation was less pronounced in Amarelle cultivars. Montmorency was the only cultivar exhibiting a slight increase in catechin concentration.

Kaempferol showed considerable cultivar- and season-dependent variation. Montmorency consistently exhibited the highest concentrations, approximately 5.4 times higher than those recorded for Szklanka z Warki. Most cultivars showed lower kaempferol levels in 2025, resulting in a seasonal average approximately 2.5 times lower than in 2024. Only Szklanka Wielka and Diemitzer Amarelle exhibited increased kaempferol concentrations during the second growing season.

Myricetin concentrations varied considerably among cultivars and between growing seasons. Łutówka consistently exhibited the highest myricetin concentrations, approximately 5.4 times higher than the lowest values recorded for Granda. Although myricetin levels in Łutówka decreased markedly in 2025, it remained one of the cultivars with the highest concentrations. Seasonal variation was more pronounced in Morello than in Amarelle cultivars, whereas Galena showed almost no year-to-year change. Increased myricetin concentrations in 2025 were observed only in Granda, Kelleris 16, and Sabina.

Quercetin concentrations exhibited marked seasonal variation. Łutówka showed the highest quercetin concentration in 2024, approximately 7.7 times higher than the lowest value recorded for Kelleris 16. Most cultivars showed substantial reductions in 2025, resulting in considerably lower variability among cultivars than in the previous growing season. No cultivar exhibited increased quercetin concentrations during the second experimental year.

Quercetin-3-*O*-rutinoside exhibited the greatest seasonal variation among the analyzed flavonoids. Łutówka and Kelleris 16 showed exceptionally high concentrations in 2024, followed by pronounced reductions in 2025. In contrast, Amarelle cultivars exhibited lower seasonal variability and generally higher relative quercetin-3-*O*-rutinoside concentrations during the second growing season than Morello cultivars.

Quercetin-3-*O*-glucoside concentrations decreased in all cultivars during the second growing season. Kelleris 16 consistently exhibited the highest quercetin-3-*O*-glucoside levels in both years, approximately 4.8 times higher than the lowest concentration recorded for Sabina. Despite the overall reduction in 2025, Kelleris 16 maintained the highest concentration, whereas cultivar differences became less pronounced.

Overall, the obtained results demonstrate significant cultivar-dependent differences in flavonoid accumulation, with Morello cultivars generally showing higher concentrations of several flavonoid compounds than Amarelle cultivars. Seasonal variation was compound-specific: quercetin-3-*O*-rutinoside, quercetin, and kaempferol showed the strongest reductions in 2025, whereas epigallocatechin and catechin displayed comparatively greater stability. The reduced variability among cultivars observed for several compounds in 2025 suggests that seasonal conditions may have influenced flavonoid accumulation to a greater extent than genotype in the second experimental year. Cultivars such as Łutówka, Kelleris 16, and Montmorency exhibited distinct flavonoid profiles, indicating their potential value for applications requiring sour cherries with high and characteristic bioactive compound profile.

The observed patterns suggest that flavonoid accumulation in tart cherry fruits is regulated by the combined effects of genotype and environmental conditions. The compound-specific responses recorded between growing seasons indicate differences in the regulation of individual branches of flavonoid biosynthesis, with some compounds appearing more environmentally responsive than others. Similar genotype × environment interactions affecting flavonoid metabolism have previously been reported for cherries and other horticultural crops [39,40,41,42].

Compared with the other flavonoids analyzed, epigallocatechin and catechin exhibited relatively small seasonal variation, suggesting greater stability of their accumulation under changing growing conditions. Similar observations have been reported in previous studies, indicating that these compounds are generally less responsive to environmental variation than other flavonoids [35,43]. As major antioxidant constituents of tart cherry fruits, they contribute significantly to the overall phytochemical profile [43].

Unlike epigallocatechin and catechin, kaempferol, myricetin, quercetin and their glycosides exhibited pronounced year-to-year variation, suggesting greater sensitivity to seasonal environmental conditions. Previous studies have shown that the accumulation of these compounds is influenced by factors such as light intensity, temperature and oxidative stress [39,40,41,43,44]. The consistent decline observed for several flavonoids in 2025, therefore, supports the important role of environmental conditions in shaping the flavonoid profile of tart cherry fruits.

The contrasting behavior of rutin and quercetin-3-*O*-glucoside further demonstrates that individual flavonol glycosides are regulated independently despite sharing common biosynthetic pathways. Previous studies have shown that environmental conditions, particularly solar radiation, water availability and temperature, can modify both the synthesis and glycosylation of flavonols, resulting in cultivar-specific accumulation patterns [42,45,46]. The persistence of characteristic flavonoid profiles across cultivars nevertheless indicates that genetic background remains the principal factor determining qualitative differences among tart cherry cultivars.

Many flavonoids identified in the present study have been reported to possess antioxidant and other biological properties [35,39,43,44,45,47,48]. However, because the present work focused exclusively on phytochemical composition, these biological activities should be regarded only as supporting background information rather than direct evidence of the functional properties of the investigated cultivars. Instead, the observed differences primarily demonstrate the considerable natural diversity of flavonoid composition among tart cherry genotypes and its modification by seasonal growing conditions.

Overall, the obtained results indicate that flavonoid accumulation is determined by the combined effects of genotype and environment, with genetic factors governing cultivar-specific flavonoid profiles and seasonal conditions modulating the accumulation of individual compounds. These findings are consistent with previous reports describing the environmental regulation of flavonoid and other phenolic compounds metabolism in cherries and other fruit crops [38,39,40,41,42,46].

### 2.4. Anthocyanins

**Figure 4:**
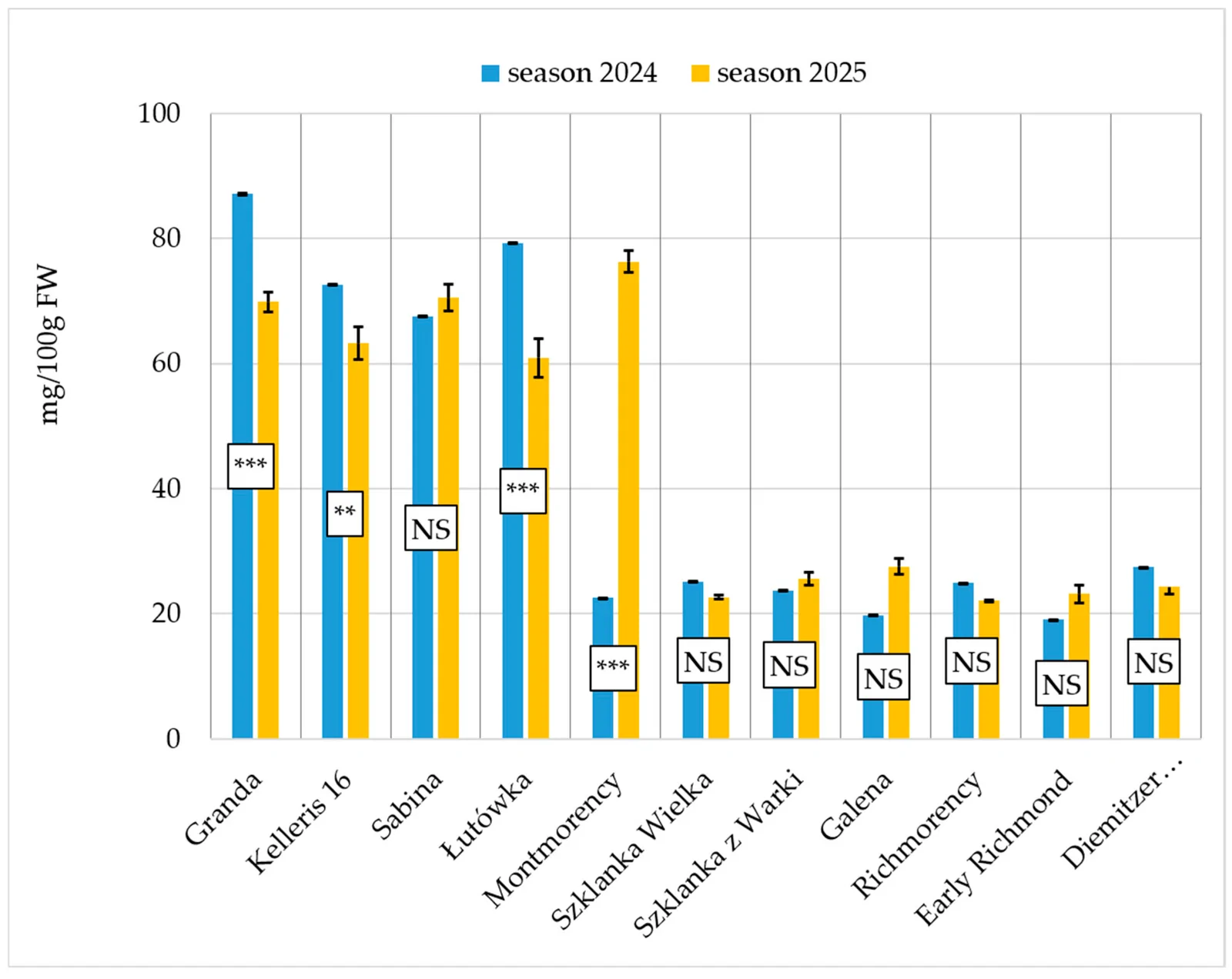
Mean values ± SE of total anthocyanin content in different tart cherry cultivars over two experimental years (2024–2025); *p*-value < 0.0001 in the ANOVA test for the influence of cultivar and cultivar × year interaction; *p*-value < 0.05 in the ANOVA test for the influence of year. Signs on the bars for each cultivar indicate significant differences between years (*p*-value (**) <0.01, (***) <0.001). NS—not significant differences.

As shown in Figure 4, the mean total anthocyanin levels across all cultivars were slightly different in 2024 compared with 2025, although individual cultivars exhibited significant differences within tart cherry groups. Anthocyanin concentrations in Morello cherries ranged between 68 mg and 78 mg per 100 g FW, while concentrations for the majority of Amarelle cherries (apart from Montmorency) ranged between 20 mg and 26 mg per 100 g FW. The Montmorency cultivar exhibited concentrations of approximately 50 mg, close to the mean value for both seasons. The highest concentrations of anthocyanins were found in Granda, and the lowest in Early Richmond.

Anthocyanins are key bioactive compounds in sour cherries, contributing to both fruit color and antioxidant and anti-inflammatory activity [22]. Higher concentrations in Morello than in most Amarelle cultivars reflect the darker pigmentation of Morello cherries. Granda, Kelleris 16, Sabina, and Łutówka are particularly suitable for fresh consumption and for use in functional foods. The slightly higher concentrations of anthocyanins in 2025, despite lower polyphenol and flavonoid levels, suggest different regulation of their biosynthesis. Similar patterns have been reported in other studies, where anthocyanins responded differently to environmental factors than other phenolics [42,49].

#### Individual Anthocyanins

As shown in Tables S3 and S4 (in the Supplementary Materials), the concentration of identified tart cherry anthocyanins varied among cultivars and between experimental years. Cyanidin-3-*O*-rutinoside showed pronounced cultivar- and season-dependent variation. In 2024, Granda and Łutówka exhibited the highest cyanidin-3-*O*-rutinoside concentrations, approximately 22- and 20-fold higher, respectively, than Early Richmond.

Most Morello cultivars maintained relatively high cyanidin-3-*O*-rutinoside levels across both seasons, whereas Amarelle cultivars displayed greater year-to-year variability. The most pronounced seasonal differences were observed in Montmorency, where cyanidin-3-*O*-rutinoside concentration was approximately 12 times higher in 2025 compared to 2024. Smaller differences were recorded in Early Richmond and Sabina, while the remaining cultivars showed relatively minor seasonal variation.

Cyanidin-3-*O*-glucoside concentrations also differed considerably among cultivars and growing seasons. Kelleris 16 consistently exhibited the highest average cyanidin-3-*O*-glucoside concentration, followed by Granda and Sabina. In 2024, the highest cyanidin-3-*O*-glucoside levels were recorded in Granda, Łutówka, and Kelleris 16, whereas Kelleris 16 and Sabina exhibited the highest concentrations in 2025. The greatest decreases were observed in Granda and Łutówka, while Galena, Montmorency, and Szklanka z Warki showed marked increases in the second compared to the first growing season.

Overall, Granda, Kelleris 16, Łutówka, and Sabina exhibited the highest anthocyanin contents among the studied cultivars, although their contributions of cyanidin-3-*O*-rutinoside and cyanidin-3-*O*-glucoside differed. In contrast, several Amarelle cultivars showed greater year-to-year variability, particularly for cyanidin-3-*O*-rutinoside.

The present study demonstrated that anthocyanin accumulation in tart cherry fruits was influenced by both cultivar and growing season. Although Morello cultivars generally accumulated higher concentrations of cyanidin derivatives than Amarelle cultivars, the magnitude and direction of seasonal changes differed considerably among cultivars, indicating that anthocyanin biosynthesis is regulated by the combined effects of genetic background and environmental conditions. Similar genotype × environment interactions have previously been reported for tart cherries and other *Prunus* species [46,50,51].

Cyanidin-3-*O*-rutinoside and cyanidin-3-*O*-glucoside are the predominant anthocyanins responsible for the characteristic red color of tart cherry fruits and contribute substantially to their antioxidant properties [38,50,52]. The consistently high concentrations of these compounds observed in fruits of cultivars such as Granda, Kelleris 16, Łutówka, and Sabina indicate their greater genetic capacity for anthocyanin accumulation, which agrees with previous reports identifying genotype as a major factor determining anthocyanin composition in sour cherries [50,51].

The contrasting seasonal responses observed among cultivars suggest that anthocyanin accumulation is strongly affected by growing conditions. In particular, the marked increase in cyanidin-3-*O*-rutinoside observed in Montmorency and the differing responses of cyanidin-3-*O*-glucoside among cultivars indicate that environmental factors may influence anthocyanin metabolism in a cultivar-specific manner. Previous studies have shown that temperature, light conditions and other environmental factors can substantially modify anthocyanin biosynthesis during fruit development [23,42,49].

Although anthocyanins are widely recognized for their antioxidant activity and their contribution to fruit quality [53], the present study was limited to the quantitative determination of individual cyanidin derivatives. Therefore, the observed differences should primarily be interpreted as evidence of cultivar- and season-dependent variation in anthocyanin composition rather than direct indicators of biological activity. Nevertheless, the distinct anthocyanin profiles identified for several cultivars provide valuable information for future studies on fruit quality, nutritional value and cultivar selection.

## 3. Materials and Methods

### 3.1. Location and Plant Material

Research work was conducted in 2024–2025. Fruits for laboratory analysis were picked from tart cherry trees growing in a collection (gene bank) located at the Experimental Orchard (central Poland, altitude 145 m above sea level, latitude 51°54″ N, longitude 20°06″ E) belonging to the National Institute of Horticultural Research in Skierniewice. One-year-old trees of tart cherry cultivars (*P. cerasus* L.), grafted onto *Prunus mahaleb* seedling rootstocks, were planted in the spring of 2017 in a grey-brown podzolic soil with a clay subsoil. Each cultivar was represented by 3 freestanding trees. During the first two years after planting the trees, the soil was kept free from weeds by mechanical cultivation. In the third year, grass was introduced in the inter-rows. During the following years, soil management included frequent grass mowing in the alleyways and maintenance of 1 m-wide herbicide strips along the tree rows. The tart cherry trees were irrigated with a drip system. Fertilization, pest and disease control were carried out in accordance with the current recommendations for commercial tart cherry orchards and the principles of integrated fruit production.

The majority of tart cherry cultivars have red skin with colored flesh and juice and are classified in the Morellos group. Some cultivars have yellow-red skin with cream flesh and transparent juice, and they belong to the Amarelle group. Four cultivars with colored juice, including: ‘Granda’, ‘Kelleris 16’, ‘Sabina’ and ‘Łutówka’ (Morello type) and seven cultivars with transparent juice, including: ‘Montmorency’, ‘Szklanka Wielka’, ‘Szklanka z Warki’, ‘Galena’, ‘Richmorency’, ‘Early Richmond’ and ‘Diemitzer Amarelle’ (Amarelle type) were used for the laboratory analysis (Table 1).

### 3.2. Weather Conditions

In 2024–2025, weather data were collected using an automatic iMetos 1 meteorological station (Pessl Instruments, Weiz, Austria) located in the Experimental Orchard in Dąbrowice at a height of 2 m above ground level. Table 2 presents monthly mean, minimum, and maximum air temperatures, together with total rainfall, from April to July, covering the key period of flowering, fruit development, and harvest.

### 3.3. Dry Matter Determination

The dry matter content of sour cherry fruits was determined by the gravimetric method according to Polish Norm [54], with slight modifications. Approximately 1 g of fresh homogenized fruit sample was weighed into previously dried and weighed glass dishes. The samples were dried in a laboratory dryer (Farma Play FP-25 W, Bielsko-Biała, Poland) at 105 °C for 12 h until constant weight was achieved. After drying, the samples were cooled in a desiccator to room temperature and weighed again. The dry matter content was calculated from the difference between the initial and final sample weight and expressed as g/100 g fresh weight (FW). All analyses were performed in triplicate.

### 3.4. Plant Material Preparation and Extraction Procedure

Tart cherry fruits were harvested at the optimum stage of technological maturity assessed on the basis of cultivar-specific skin color, full fruit coloring, fruit size, and suitability for processing. For each cultivar, fruits were collected from three individual trees. A total of 2 kg of fully colored fruits was harvested per cultivar, with fruits taken from different parts of the tree canopy, including the upper, lower, inner, and outer sections. For each cultivar and growing season, fruits were collected from three trees grown under identical orchard conditions. Equal amounts of fruits from the three trees were pooled to obtain one representative composite sample for each cultivar. The composite sample was then analyzed using three independent laboratory (analytical) replicates for all chemical determinations to assess the repeatability of the analytical procedure. The mean values obtained from the analytical replicates were used for statistical analysis. Because the fruits from the three trees were pooled before analysis, the analytical replicates do not represent independent biological replicates. Immediately after harvest, the samples were transported from the orchard to the laboratory in cooled polystyrene boxes.

The fruits were thoroughly washed and air-dried. The stones and fruit stalks were then removed. Subsequently, the prepared fruits were freeze-dried for 72 h using a Labconco FreeZone 2.5 freeze dryer (Labconco Corporation, Kansas City, MO, USA) at −40 °C and a pressure of 0.100 mbar. The freeze-dried material was ground into a fine powder using a laboratory mill (IKA A11, Staufen, Germany) and stored at −80 °C until analysis.

### 3.5. HPLC Analysis of Polyphenolic Compounds

Polyphenols in tart cherry fruits were determined by high-performance liquid chromatography (HPLC) according to the procedure described previously [49], with the following analytical conditions: 100 mg of freeze-dried fruit powder was transferred into a polypropylene laboratory tube and extracted with 5 mL of 80% methanol. After thorough mixing with a vortex mixer, the samples were sonicated in an ultrasonic bath (6000 Hz) at 30 °C for 10 min. The extracts were subsequently clarified by two consecutive centrifugation steps, first at 6000 rpm for 10 min and then at 12,000 rpm for 5 min. Following centrifugation, 1 mL of the clear supernatant was collected and subjected to HPLC analysis using an injection volume of 50 µL. Chromatographic separation was carried out using a Shimadzu HPLC system (Shimadzu USA Manufacturing Inc., Canby, OR, USA) consisting of two LC-20AD pumps, a SIL-20AC autosampler, a CMB-20A system controller, an SPD-20AV UV–Vis detector, and a CTO-20AC column oven. Separation was performed on a Phenomenex Fusion-RP 80 Å column (250 mm × 4.60 mm). The mobile phase consisted of solvent A (10% acetonitrile) and solvent B (55% acetonitrile), both adjusted to pH 3.0 with orthophosphoric acid. Elution was achieved using the following gradient program: 0.00–23.00 min, 95% A/5% B; 23.01–27.00 min, 50% A/50% B; 27.01–29.00 min, 20% A/80% B; 29.01–36.00 min, 20% A/80% B; and 36.01–42.00 min, 95% A/5% B. The total chromatographic run time was 42 min at a flow rate of 1.0 mL min^−1^, while detection was performed over a wavelength range of 250–370 nm. Identification of individual phenolic compounds was based on comparisons with authentic external standards obtained from Fluka and Sigma-Aldrich. The results were expressed as mg/100 g FW.

### 3.6. HPLC Analysis of Anthocyanins

Anthocyanins were analyzed by HPLC according to the previously described method [55]. The initial extraction procedure was identical to that used for polyphenol analysis and is described in Section 3.5 (HPLC Analysis of Polyphenolic Compounds). Briefly, freeze-dried samples were extracted with 80% methanol, and the resulting extract was used for further anthocyanin determination. For sample preparation, 2.5 mL of the obtained supernatant was transferred into a clean (orange) polypropylene tube, followed by the addition of 2.5 mL of 1.0 M HCl and 5.0 mL of pure methanol. The mixture was cooled and kept at 5 °C for 10 min (in the dark). Subsequently, 50 µL of the prepared extract was injected into the HPLC system for chromatographic analysis. Anthocyanin separation was carried out under isocratic conditions using a mobile phase composed of 5% acetic acid, acetonitrile, and methanol (70:10:20, *v*/*v*/*v*). The chromatographic analysis was performed at a flow rate of 1.0 mL min^−1^, and detection was performed at a wavelength of 530 nm. Individual anthocyanins were identified by comparing their retention times with those of authentic reference standards. Cyanidin-3-*O*-glucoside chloride (CAS No. 7084-24-4, Sigma-Aldrich, Merck, Germany; Product No. PHL89616) and cyanidin-3-*O*-rutinoside chloride (CAS No. 18719-76-1, Sigma-Aldrich, Merck, Germany; Product No. PHL80577) were used as external reference standards. Quantification was performed using separate five-point external calibration curves prepared for each anthocyanin standard. The concentrations determined in freeze-dried samples were recalculated to a fresh weight basis and expressed as mg/100 g fresh weight (FW) in all tables and figures.

### 3.7. Statistical Analysis

Chemical analyses were subsequently performed using three laboratory (analytical) replicates for each composite biological sample, and the obtained results are presented as mean values ± standard error (SE). The analytical replicates were used to assess the repeatability of the analytical procedure. Statistical analyses were conducted using Statgraphics Centurion software (version 15.2.11.0, StatPoint Technologies Inc., Warrenton, VA, USA). The effects of cultivar and growing season (year) were evaluated using two-way analysis of variance (ANOVA), followed by Tukey’s honestly significant difference *post hoc* test for pairwise comparisons among treatment means when significant main effects or interactions were detected. Differences were considered statistically significant at *p* < 0.05. Prior to ANOVA, the data were examined for compliance with the assumptions of normality using the Shapiro–Wilk test.

## 4. Conclusions

This study demonstrated significant cultivar- and season-dependent differences in the accumulation of dry matter and bioactive compounds in tart cherry (*Prunus cerasus* L.) fruits. Among the evaluated cultivars, Kelleris 16 and Łutówka exhibited the most favorable phytochemical profiles, combining high concentrations of polyphenols, flavonoids, and anthocyanins, while Szklanka Wielka was distinguished by its particularly high levels of chlorogenic and caffeic acids. Although seasonal conditions influenced the concentrations of most phenolic compounds, cultivar-related differences remained the primary factor determining fruit phytochemical quality.

From a practical perspective, obtaining tart cherry fruits with different phytochemical profiles may be relevant for nutritional quality and technological applications. The obtained results may support cultivar selection according to the intended use of tart cherries. Morello cultivars, particularly Łutówka, Kelleris 16, and Sabina, were characterized by high concentrations of flavonoids and anthocyanins, making them especially suitable for the production of juices, concentrates, frozen products, and other processed foods where intense color and high contents of phenolic compounds are desirable. In contrast, selected Amarelle cultivars, particularly Szklanka Wielka, were distinguished by relatively high concentrations of chlorogenic and caffeic acids and may represent valuable options for fresh consumption and for the development of processed products with diverse phenolic profiles. These findings provide practical guidance for growers, processors, and consumers seeking cultivars with specific nutritional and technological characteristics. Moreover, these findings also provide valuable information for growers, breeders, processors, and retailers seeking to align tart cherry production with consumer expectations for high-quality fruits rich in natural bioactive compounds. The identification of cultivars with superior phytochemical profiles may inform cultivar selection strategies to increase the added value and market competitiveness of tart cherry products.

Future research should focus on evaluating the biological activity of the most promising tart cherry cultivars using in vitro models to determine whether the favourable phytochemical profiles observed in this study translate into measurable biological effects. Further studies on the bioavailability of key phenolic compounds and validation under different growing conditions would also contribute to a better understanding of their nutritional and potential functional significance.

## 5. Limitations

The present study has several limitations that should be considered when interpreting the results. First, fruits were collected from three trees per cultivar and pooled to obtain one representative composite sample for each cultivar and growing season. Consequently, although the analytical determinations were performed in triplicate to ensure the repeatability of the measurements, the study design did not allow the assessment of tree-to-tree biological variability within cultivars. Second, all cultivars were evaluated at a single experimental location over two consecutive growing seasons; therefore, genotype × environment interactions under different climatic and agronomic conditions require further investigation. Finally, although the phytochemical composition of the fruits was comprehensively characterized, the present study was limited to compositional analyses, and the in vitro biological activity of the identified compounds was not evaluated. Future studies should address these limitations to provide a more comprehensive assessment of the nutritional and functional potential of tart cherry cultivars.

## Figures and Tables

**Table 1:** Selected characteristics of the 11 tart cherry cultivars studied.

Cultivar	Country of Origin	Ripening Time	Fruit Weight [g]	Juice Color
Granda	Poland	I dec. VII	5.0–7.0	red
Kelleris 16	Denmark	II dec. VII	5.0–5.5	red
Sabina	Poland	I dec. VII	4.5–5.5	red
Łutówka (English Morello type)	Unknown	e. VII	5.5–6.0	red
Montmorency	USA	I-II dec. VII	4.0–5.0	colorless
Szklanka Wielka	Poland	e. VI	4.0–5.0	colorless
Szklanka z Warki	Poland	e. VI	4.0–4.5	colorless
Galena	Poland	e. VI	6.0–7.5	colorless
Richmorency	USA	I dec. VII	4.5–5.0	colorless
Early Richmond	USA	e. VI	4.5–5.0	colorless
Diemitzer Amarelle	Germany	I dec. VII	4.0–5.0	colorless

**Table 2:** Weather conditions at the Experimental Orchard in Dąbrowice. Monthly averages, maximum and minimum air temperatures (°C) and total monthly rainfall (mm) from April to July 2024–2025.

Months/Year	Air Temperature (°C)			Rainfall (mm)
	Average	Maximum	Minimum	Total
April/2024	10.84	28.23	−1.87	18.8
May/2024	16.31	28.48	−0.69	46.8
June/2024	18.95	33.65	6.07	43.4
July/2024	21.16	35.07	8.45	21.4
April/2025	10.79	27.95	−3.43	26.2
May/2025	11.16	26.18	−1.23	55.0
June/2025	17.86	31.54	5.63	38.4
July/2025	19.07	35.97	6.94	106.4

## Data Availability

The original contributions presented in this study are included in the article. Further inquiries can be directed to the corresponding author.

## Supplementary Materials

The following supporting information can be downloaded at https://www.mdpi.com/article/10.3390/molecules31162881/s1, Table S1: The mean values of dry matter = DM (g/100 g FW ± SE) and phenolic acids-gallic acid (GA), *p*-coumaric acid (*p*-CA), ferulic acid (FA), chlorogenic acid (CGA), caffeic acid (CafA), and *trans*-cinnamic acid (*t*-CA) (mg/100 g FW ± SE) in examined tart cherry cultivars in 2024–2025; Table S2: The dry matter = DM (g/100 g FW ± SE) and phenolic acids-gallic acid (GA), p-coumaric acid (p-CA), ferulic acid (FA), chlorogenic acid (CGA), caffeic acid (CafA), and *trans*-cinnamic acid (t-CA) (mg/100 g FW ± SE) in examined tart cherry cultivars in season 2024 and season 2025; Table S3: The mean values of flavonoids: epigallocatechin (EGC), catechin (CAT), kaempferol (KAE), myricetin (MYR), quercetin (QUE), quercetin-3-*O*-rutinoside (RUT), quercetin-3-*O*-glucoside (QG), cyanidin-3-*O*-rutinoside (C3R), and cyanidin-3-*O*-glucoside (C3G) (mg/100 g FW ± SE) in examined tart cherry cultivars in both experimental years; Table S4: The mean values of flavonoids: epigallocatechin (EGC), catechin (CAT), kaempferol (KAE), myricetin (MYR), quercetin (QUE), quercetin-3-*O*-rutinoside (RUT), quercetin-3-*O*-glucoside (QG), cyanidin-3-*O*-rutinoside (C3R), and cyanidin-3-*O*-glucoside (C3G) (mg/100 g FW ± SE) in examined tart cherry cultivars in two experimental years: 2024 and 2025. Table S5: Validation parameters of the HPLC method, including LOD, LOQ, recovery, precision (%RSD), linearity (R^2^) and wavelength for the analyzed phenolic standards.

## Abbreviations

The following abbreviations are used in this manuscript:

C3G	Cyanidin-3-*O*-glucoside
C3R	Cyanidin-3-*O*-rutinoside
CafA	Caffeic acid
CAT	Catechin
CGA	Chlorogenic acid
DM	Dry matter
EGC	Epigallocatechin
FA	Ferulic acid
FW	Fresh weight
GA	Gallic acid
GAE	Gallic acid equivalents
KAE	Kaempferol
MYR	Myricetin
*p*-CA	*p*-Coumaric acid
QG	Quercetin-3-*O*-glucoside
QUE	Quercetin
RUT	Quercetin-3-*O*-rutinoside
SE	Standard Error
*t*-CA	*trans*-Cinnamic acid
