# Supplementary material for: Polyphenol and Flavonoid Levels of Selected Morello and Amarelle Tart Cherry Cultivars (Prunus cerasus L.): Implications for Orchard Production and Consumer Selection
Source: Molecules. 2026 Aug 18;31(16):2881. doi: 10.3390/molecules31162881 (PMC13515572; doi:10.3390/molecules31162881)
Supplement: Supplementary file 1 [file molecules-31-02881-s001.zip › molecules-4403211-supplementary.pdf]

Supplementary Material to the Article:

Polyphenol and Flavonoid Levels of Selected Morello and Amarelle Tart Cherry Cultivars (*Prunus cerasus* L.): Implications for Orchard Production and Consumer Selection

Justyna Traore [1], Elżbieta Rozpara [2], Agnieszka Głowacka [2], Witold Danelski [2], Ewelina Hallmann [1,3], Alicja Ponder [1], Małgorzata Żebrowska-Krasuska [1], Klaudia Kopczyńska [1], Dominika Średnicka-Tober [1], Renata Kazimierczak [1,*]

1 Department of Functional and Organic Food, Institute of Human Nutrition Sciences, Warsaw University of Life Sciences, Warsaw, Poland; justynatraore@gmail.com (J.T.), ewelina_hallmann@sggw.edu.pl (E.H.), alicja_ponder@sggw.edu.pl (A.P.), malgorzata_zebrowska@sggw.edu.pl M.Ż.-K.), klaudia_kopczynska@sggw.edu.pl (K.K.); dominika_srednicka_tober@sggw.edu.pl (D.Ś.-T.); renata_kazimierczak@sggw.edu.pl (R.K.)

2 Cultivar Testing, Nursery and Gene Bank Resources Department, The National Institute of Horticultural Research, Skierniewice, Poland; elzbieta.rozpara@inhort.pl (E.R.); agnieszka.glowacka@inhort.pl (A.G.); witold.danelski@inhort.pl (W.D.)

3 Bioeconomy Research Institute, Agriculture Academy, Vytautas Magnus University, Donelaicio 58, 44248 Kaunas, Lithuania; ewelina_hallmann@sggw.edu.pl (E.H.)

* Correspondence: renata_kazimierczak@sggw.edu.pl (R.K)

Table S1. The mean values of dry matter = DM (g/100 g FW ± SE) and phenolic acids - gallic acid (GA), *p*-coumaric acid (*p*-CA), ferulic acid (FA), chlorogenic acid (CGA), caffeic acid (CafA), and *trans*-cinnamic acid (*t*-CA) (mg/100 g FW ± SE) in examined tart cherry cultivars in 2024-2025.

**Category**	**Cultivar**	**DM**	**GA**	***p*-CA**	**FA**	**CGA**	**CafA**	***t*-CA**
cultivar average	Granda	12.29±0.22^{c}	6.04±1.05^{b}	8.22±1.17^{b}	2.63±0.70^{a}	3.92±0.15^{d}	0.34±0.04^{d}	0.16±0.04^{b}
	Kelleris 16	14.06±0.36^{a}	6.99±2.45^{b}	24.29±7.83^{a}	1.46±0.08^{b}	5.34±0.18^{bc}	0.38±0.04^{d}	0.15±0.01^{b}
	Sabina	13.33±0.42^{b}	5.42±1.48^{c}	6.95±1.26^{c}	1.13±0.09^{bc}	6.97±0.17^{b}	0.31±0.02^{d}	0.14±0.03^{b}
	Łutówka	12.62±0.34^{c}	9.11±1.82^{a}	11.05±3.70^{a}	1.60±0.20^{b}	4.36±0.17^{c}	0.40±0.02^{c}	0.34±0.17^{a}
	Montmorency	12.39±0.21^{c}	6.31±0.17^{b}	6.40±0.83^{c}	1.37±0.11^{b}	4.72±0.41^{c}	1.53±0.10^{a}	0.38±0.02^{a}
	Szklanka Wielka	13.18±0.43^{b}	5.83±0.48^{c}	9.06±2.21^{a}	1.14±0.12^{bc}	7.92±0.19^{a}	1.84±0.03^{a}	0.12±0.01^{b}
	Szklanka z Warki	12.05±0.38^{c}	4.68±0.41^{d}	8.83±1.86^{b}	2.15±0.30^{a}	3.68±0.21^{d}	1.13±0.08^{a}	0.14±0.01^{b}
	Galena	11.64±0.37^{d}	2.96±0.71^{f}	5.17±0.71^{d}	0.88±0.27^{d}	4.18±0.26^{c}	0.65±0.04^{c}	0.19±0.01^{b}
	Richmorency	11.07±0.20^{d}	5.22±0.59^{c}	6.73±0.18^{c}	0.79±0.15^{d}	5.30±0.58^{b}	1.62±0.03^{a}	0.07±0.01^{c}
	Early Richmond	11.58±0.37^{d}	4.61±0.15^{d}	7.82±1.62^{b}	2.19±0.40^{a}	2.73±0.20^{d}	0.50±0.03^{c}	0.15±0.01^{b}
	Diemitzer Ama-relle	12.43±0.26^{c}	3.77±1.29^{e}	10.74±2.03^{a}	1.29±0.13^{bc}	5.79±0.33^{b}	0.83±0.07^{b}	0.11±0.01^{b}
season average	2024	12.14±0.13^{B}	7.94±0.36^{A}	14.56±1.24^{A}	1.82±0.14^{A}	5.22±0.19^{A}	0.83±0.06^{B}	0.22±0.03^{A}
	2025	12.70±0.14^{A}	3.14±0.22^{B}	4.57±0.11^{B}	1.21±0.04^{B}	4.77±0.20^{B}	0.91±0.07^{A}	0.14±0.01^{B}
p-value	cultivar	<0.0001	<0.0001	<0.0001	<0.0001	<0.0001	<0.0001	0.0005
	season	0.0042	<0.0001	<0.0001	<0.0001	0.0004	<0.0001	0.0103

*Data is presented as the mean ± SE with ANOVA p-value; Means in columns followed by the different letters are significantly different at the 5% level of probability ($p<0.05$).

Table S2. The dry matter = DM (g/100 g FW ± SE) and phenolic acids - gallic acid (GA), *p-c*oumaric acid (*p*-CA), ferulic acid (FA), chlorogenic acid (CGA), caffeic acid (CafA), and trans-cinnamic acid (*t*-CA) (mg/100 g FW ± SE) in examined tart cherry cultivars in season 2024 and season 2025.

Category	Cultivar	DM	GA	*p*-CA	FA	CGA	CafA	*t*-CA
season 2024	Granda	11.94±0.10^{e}	9.80±0.28^{b}	11.95±0.34^{c}	4.12±0.06^{a}	4.37±0.21^{cd}	0.38±0.01^{d}	0.23±0.07^{b}
	Kelleris 16	13.58±0.12^{b}	12.99±0.02^{a}	43.47±0.15^{a}	1.47±0.01^{d}	5.60±0.22^{c}	0.46±0.02^{d}	0.14±0.01^{c}
	Sabina	12.30±0.10^{c}	9.03±0.01^{b}	10.04±0.09^{c}	0.91±0.01^{e}	7.26±0.15^{a}	0.35±0.02^{d}	0.16±0.01^{c}
	Łutówka	13.03±0.07^{b}	13.55±0.04^{a}	18.19±4.54^{b}	2.07±0.06^{c}	4.60±0.15^{e}	0.45±0.02^{d}	0.41±0.01^{b}
	Montmorency	11.96±0.07^{e}	6.41±0.03^{c}	8.37±0.34^{d}	1.64±0.02^{d}	5.62±0.12^{c}	1.30±0.03^{ab}	0.68±0.24^{a}
	Szklanka Wielka	13.47±0.83^{b}	6.88±0.42^{c}	13.68±2.29^{bc}	0.91±0.14^{e}	7.50±0.17^{a}	1.89±0.04^{a}	0.13±0.01^{c}
	Szklanka z Warki	11.55±0.28^{e}	5.67±0.01^{c}	13.38±0.05^{bc}	2.89±0.01^{c}	3.60±0.18^{e}	0.95±0.01^{b}	0.15±0.01^{c}
	Galena	10.97±0.15^{e}	4.70±0.10^{d}	6.58±0.71^{f}	0.95±0.53^{e}	4.55±0.20^{cd}	0.56±0.02^{c}	0.18±0.01^{c}
	Richmorency	10.61±0.13^{e}	6.66±0.02^{c}	7.10±0.01^{e}	0.90±0.01^{e}	6.71±0.15^{b}	1.56±0.02^{a}	0.07±0.01^{d}
	Early Richmond	11.25±0.15^{e}	4.74±0.01^{d}	11.75±0.02^{c}	3.16±0.01^{b}	2.43±0.23^{f}	0.55±0.02^{c}	0.14±0.01^{c}
	Diemitzer Amarelle	12.83±0.13^{c}	6.92±0.01^{c}	15.70±0.06^{b}	0.98±0.02^{e}	5.16±0.07^{c}	0.67±0.02^{c}	0.09±0.01^{d}
season 2025	Granda	12.64±0.15^{c}	2.29±1.32^{ef}	4.48±3.06^{g}	1.13±0.07^{d}	3.46±0.43^{e}	0.29±0.10^{e}	0.08±0.01^{d}
	Kelleris 16	14.54±0.04^{a}	1.00±1.27^{f}	5.11±3.10^{ef}	1.45±0.07^{d}	5.08±0.50^{c}	0.29±0.09^{e}	0.16±0.01^{c}
	Sabina	14.35±0.18^{a}	1.82±0.10^{f}	3.87±0.19^{g}	1.35±0.03^{d}	6.68±0.16^{b}	0.27±0.01^{e}	0.13±0.03^{c}
	Łutówka	12.21±0.19^{c}	4.66±0.32^{d}	3.90±0.35^{g}	1.13±0.20^{d}	4.13±0.58^{d}	0.36±0.02^{e}	0.27±0.02^{b}
	Montmorency	12.82±0.19^{c}	6.21±0.33^{c}	4.43±0.27^{g}	1.11±0.19^{d}	3.83±0.41^{e}	1.77±0.02^{a}	0.08±0.01^{d}
	Szklanka Wielka	12.89±0.85^{c}	4.77±0.11^{d}	4.44±0.28^{g}	1.38±0.15^{d}	8.34±0.18^{a}	1.79±0.01^{a}	0.11±0.01^{c}
	Szklanka z Warki	12.54±0.79^{c}	3.69±0.16^{e}	4.29±0.28^{g}	1.42±0.05^{d}	3.75±0.49^{e}	1.31±0.01^{ab}	0.14±0.01^{c}
	Galena	12.30±0.60^{c}	1.21±0.22^{f}	3.76±0.45^{h}	0.81±0.03^{e}	3.80±0.53^{e}	0.75±0.01^{c}	0.21±0.01^{c}
	Richmorency	11.53±0.11^{d}	3.79±0.69^{d}	6.35±0.18^{f}	0.67±0.06^{f}	3.89±0.20^{e}	1.68±0.01^{a}	0.08±0.01^{d}
	Early Richmond	11.91±0.10^{d}	4.47±0.70^{d}	3.90±0.15^{h}	1.21±0.09^{d}	3.03±0.82^{e}	0.46±0.02^{d}	0.15±0.03^{c}
	Diemitzer Amarelle	12.02±0.77^{c}	0.62±0.02^{g}	5.78±0.18^{ef}	1.59±0.07^{d}	6.42±0.66^{b}	0.99±0.03^{b}	0.13±0.04^{c}
p-value	cultivar x season	0.0367	<0.0001	<0.0001	<0.0001	<0.0001	<0.0001	0.0009

*Data is presented as the mean ± SE with ANOVA p-value; Means in columns followed by the different letters are significantly different at the 5% level of probability (p<0.05).

Table S3. The mean values of flavonoids: epigallocatechin (EGC), catechin (CAT), kaempferol (KAE), myricetin (MYR), quercetin (QUE), quercetin-3-O-rutinoside (RUT), quercetin-3-O-glucoside (QG), cyanidin-3-O-rutinoside (C3R), and cyanidin-3-O-glucoside (C3G) (mg/100 g FW ± SE) in examined tart cherry cultivars in both experimental years.

Category	Cultivar	EGC	CAT	KAE	MYR	QUE	RUT	QG	C3R	C3G
cultivar average	Granda	4.02±0.68^{a}	9.21±0.66^{b}	2.14±0.48^{c}	0.19±0.06^{d}	0.45±0.06^{b}	6.18±1.27^{b}	0.55±0.06^{c}	48.18±10.11^{a}	30.31±3.64^{a}
	Kelleris 16	3.81±1.19^{b}	11.34±1.76^{a}	2.60±0.71^{c}	0.27±0.03^{c}	0.28±0.01^{d}	11.32±3.97^{a}	1.82±0.15^{a}	36.49±2.25^{b}	31.42±0.68^{a}
	Sabina	4.87±0.29^{a}	7.14±0.66^{c}	1.38±0.23^{d}	0.23±0.02^{c}	0.42±0.08^{b}	6.53±2.07^{b}	0.38±0.08^{d}	40.61±0.83^{a}	28.44±0.42^{a}
	Łutówka	5.17±0.19^{a}	14.75±2.13^{a}	1.04±0.10^{d}	1.03±0.27^{a}	1.32±0.47^{a}	11.76±3.58^{a}	0.53±0.09^{c}	42.97±2.39^{a}	27.12±1.69^{ab}
	Montmorency	1.86±0.26^{c}	6.70±0.11^{cd}	5.05±1.24^{a}	0.53±0.06^{b}	0.36±0.07^{c}	4.71±0.70^{c}	0.57±0.05^{c}	27.95±9.64^{c}	21.49±1.38^{b}
	Szklanka Wielka	1.20±0.04^{c}	5.89±1.17^{d}	2.04±0.07^{c}	0.44±0.04^{b}	0.42±0.08^{b}	3.83±0.48^{cd}	0.60±0.09^{c}	3.94±0.27^{e}	19.95±0.28^{bc}
	Szklanka z Warki	1.28±0.12^{c}	4.86±0.29^{e}	0.93±0.11^{e}	0.36±0.03^{c}	0.44±0.13^{b}	2.85±0.76^{d}	0.46±0.04^{c}	3.86±0.74^{e}	20.78±1.20^{b}
	Galena	1.17±0.07^{c}	7.97±0.59^{c}	3.23±1.09^{b}	0.37±0.06^{c}	0.38±0.06^{c}	2.32±0.27^{d}	0.73±0.11^{b}	2.82±0.26^{f}	20.87±1.87^{b}
	Richmorency	1.17±0.03^{c}	4.41±0.70^{e}	1.86±0.26^{d}	0.42±0.04^{b}	0.86±0.28^{a}	4.46±0.55^{c}	0.71±0.14^{b}	6.00±0.65^{d}	17.49±0.09^{c}
	Early Richmond	0.88±0.02^{d}	4.75±0.10^{e}	2.09±0.17^{c}	0.49±0.06^{b}	0.30±0.05^{c}	3.93±0.24^{cd}	0.73±0.12^{b}	3.60±0.52^{e}	17.55±0.62^{c}
	Diemitzer Amarelle	1.11±0.05^{c}	6.94±0.64^{a}	1.51±0.13^{d}	0.47±0.02^{b}	0.39±0.11^{bc}	4.42±0.66^{c}	0.58±0.18^{c}	6.18±0.43^{d}	19.68±0.45^{bc}
season average	2024	2.26±0.20^{B}	9.57±0.55^{A}	3.12±0.26^{A}	0.53±0.05^{A}	0.83±0.07^{A}	9.11±0.80^{A}	1.02±0.09^{A}	19.81±2.44^{B}	22.85±0.79^{B}
	2025	2.57±0.21^{A}	5.70±0.24^{B}	1.22±0.07^{B}	0.34±0.01^{B}	0.19±0.01^{B}	2.22±0.12^{B}	0.37±0.01^{B}	20.67±2.37^{A}	23.53±0.57^{A}
p-value	cultivar	< 0.0001	< 0.0001	< 0.0001	< 0.0001	< 0.0001	< 0.0001	< 0.0001	< 0.0001	< 0.0001
	season	< 0.0001	< 0.0001	< 0.0001	< 0.0001	< 0.0001	< 0.0001	< 0.0001	0.032	0.027

*Data is presented as the mean ± SE with ANOVA p-value; Means in columns followed by the different letters are significantly different at the 5% level of probability ($p < 0.05$).

Table S4. The mean values of flavonoids: epigallocatechin (EGC), catechin (CAT), kaempferol (KAE), myricetin (MYR), quercetin (QUE), quercetin-3-O-rutinoside (RUT), quercetin-3-O-glucoside (QG), cyanidin-3-O-rutinoside (C3R), and cyanidin-3-O-glucoside (C3G) (mg/100 g FW ± SE) in examined tart cherry cultivars in two experimental years: 2024 and 2025.

Category	Cultivar	EGC	CAT	KAE	MYR	QUE	RUT	QG	C3R	C3G
	Granda	4.33±0.14^{b}	11.74±0.83^{a}	3.68±0.02^{c}	0.08±0.01^{e}	0.71±0.01^{c}	9.17±0.13^{b}	0.74±0.03^{c}	52.85±0.13^{a}	34.27±0.08^{a}
	Kelleris 16	4.21±0.02^{b}	15.61±0.03^{a}	4.33±0.20^{b}	0.20±0.01^{e}	0.32±0.01^{e}	21.05±0.04^{a}	3.17±0.04^{a}	41.75±0.09^{ab}	30.85±0.02^{a}
	Sabina	4.21±0.01^{b}	8.76±0.01^{bc}	1.93±0.13^{de}	0.18±0.01^{e}	0.61±0.01^{d}	11.60±0.01^{b}	0.57±0.01^{d}	39.92±0.02^{b}	27.64±0.01^{b}
	Łutówka	4.74±0.01^{b}	19.93±0.02^{a}	1.28±0.07^{e}	1.70±0.03^{a}	2.46±0.03^{a}	22.98±0.05^{a}	0.73±0.03^{c}	48.23±0.03^{a}	31.02±0.02^{a}
	Montmorency	1.23±0.01^{e}	6.53±0.03^{c}	7.97±0.65^{a}	0.69±0.01^{b}	0.53±0.01^{d}	6.43±0.08^{c}	0.69±0.05^{c}	4.41±0.01^{c}	18.14±0.01^{c}
season 2024	Szklanka Wielka	1.24±0.07^{e}	8.39±1.14^{bc}	1.94±0.12^{de}	0.52±0.01^{b}	0.62±0.02^{d}	5.00±0.04^{d}	0.78±0.11^{c}	4.56±0.02^{c}	20.60±0.01^{c}
	Szklanka z Warki	0.99±0.01^{e}	5.56±0.01^{d}	1.18±0.08^{e}	0.43±0.02^{c}	0.73±0.01^{c}	4.72±0.01^{d}	0.53±0.04^{d}	5.67±0.0^{c}	18.05±0.01^{c}
	Galena	0.99±0.01^{e}	9.31±0.28^{b}	5.87±0.37^{b}	0.37±0.01^{c}	0.52±0.01^{d}	2.97±0.05^{f}	0.99±0.04^{b}	3.34±0.01^{d}	16.46±0.01^{e}
	Richmorency	1.10±0.01^{e}	6.13±0.04^{c}	2.47±0.14^{d}	0.50±0.01^{b}	1.54±0.01^{b}	5.78±0.03^{d}	1.05±0.04^{b}	7.59±0.01^{c}	17.34±0.01^{dj}
	Early Richmond	0.83±0.01^{f}	4.83±0.01^{e}	2.48±0.08^{d}	0.63±0.01^{b}	0.41±0.01^{de}	4.49±0.01^{d}	1.01±0.08^{b}	2.42±0.01^{e}	16.68±0.01^{e}
	Diemitzer Ama-relle	1.00±0.01^{f}	8.46±0.01^{bc}	1.20±0.02^{e}	0.51±0.01^{b}	0.65±0.01^{c}	6.04±0.03^{c}	0.99±0.09^{b}	7.15±0.01^{c}	20.29±0.01^{c}
	Granda	3.71±0.69^{c}	6.67±0.54^{c}	0.61±0.16^{g}	0.31±0.06^{c}	0.18±0.06^{g}	3.19±0.77^{e}	0.36±0.20^{e}	43.51±9.30^{a}	26.34±1.30^{b}
	Kelleris 16	3.41±0.71^{c}	7.06±0.52^{c}	0.87±0.16^{f}	0.34±0.06^{c}	0.25±0.06^{f}	1.59±0.75^{g}	0.47±0.13^{d}	31.24±9.90^{bc}	31.99±1.78^{a}
	Sabina	5.53±0.10^{a}	5.51±0.09^{d}	0.82±0.02^{f}	0.29±0.01^{d}	0.23±0.01^{f}	1.45±0.04^{g}	0.20±0.01^{f}	41.29±0.96^{ab}	29.24±0.63^{a}
	Łutówka	5.61±0.20^{a}	9.58±0.35^{b}	0.80±0.10^{f}	0.37±0.02^{c}	0.18±0.02^{g}	0.53±0.46^{h}	0.32±0.04^{f}	37.71±2.77^{b}	23.21±2.24^{bc}
	Montmorency	2.49±0.21^{d}	6.88±0.50^{c}	2.13±0.08^{d}	0.37±0.03^{c}	0.19±0.03^{g}	2.99±0.42^{f}	0.45±0.03^{de}	51.50±3.41^{a}	24.83±2.11^{bc}
season 2025	Szklanka Wielka	1.17±0.18^{e}	3.40±0.51^{e}	2.14±0.05^{d}	0.36±0.03^{c}	0.22±0.03^{f}	2.67±0.03^{f}	0.42±0.02^{e}	3.32±1.33^{d}	19.31±1.28^{c}
	Szklanka z Warki	1.57±0.48^{de}	4.16±0.42^{e}	0.67±0.02^{g}	0.29±0.01^{d}	0.14±0.01^{h}	0.99±0.08^{h}	0.40±0.07^{e}	2.05±0.17^{e}	23.51±0.68^{bc}
	Galena	1.35±0.40^{e}	6.64±0.45^{c}	0.60±0.01^{g}	0.36±0.01^{c}	0.23±0.01^{f}	1.66±0.07^{g}	0.47±0.07^{d}	2.30±0.39^{e}	25.28±0.69^{b}
	Richmorency	1.25±0.19^{e}	2.69±0.05^{f}	1.25±0.01^{e}	0.33±0.01^{c}	0.17±0.01^{g}	3.13±0.05^{e}	0.37±0.01^{f}	4.41±1.57^{c}	17.64±0.53^{d}
	Early Richmond	0.93±0.17^{f}	4.67±0.90^{e}	1.70±0.02^{de}	0.35±0.01^{c}	0.18±0.02^{g}	3.36±0.29^{e}	0.45±0.03^{de}	4.78±1.57^{c}	18.42±2.14^{cd}
	Diemitzer Ama-relle	1.23±0.17^{e}	5.41±1.33^{d}	1.83±0.04^{de}	0.43±0.01^{c}	0.12±0.04^{h}	2.80±0.27^{f}	0.16±0.04^{g}	5.22±1.33^{c}	19.08±1.77^{c}
p-value	cultivar × season	< 0.0001	< 0.0001	< 0.0001	< 0.0001	< 0.0001	< 0.0001	< 0.0001	< 0.0001	< 0.0001

*Data is presented as the mean ± SE with ANOVA p-value; Means in columns followed by the different letters are significantly different at the 5% level of probability ($p < 0.05$).

Table S5. Validation parameters of the HPLC method, including LOD, LOQ, recovery, precision (%RSD), linearity (R^2) and wavelength for the analyzed phenolic standards.

Phenolic groups	compounds (standards)	LOD	LOQ	% recov-ery	% RSD for peak area	R^2	wavelength (nm)
Hydroxybenzoic ac-ids	Gallic acid	0.15	0.32	99.16	1.95	0.9997	250
Hydroxycinaminic acids	Chlorogenic acid	0.60	1.80	97.11	1.25	0.9988	250
	Caffeic acid	0.05	0.15	99.99	0.89	0.9999	250
	p-Coumaric acid	0.62	1.85	99.86	1.73	0.9984	250
	Ferulic acid	0.15	0.45	99.87	1.55	0.9999	250
	trans-Cinnaminic acid	0.016	0.05	99.97	0.89	0.9999	250
Flavan-3-ols	Epigallocatechin	0.22	0.65	99.25	0.45	0.9999	270
	Catechin	0.65	2.30	99.90	0.71	0.9998	270
Flavonols	Quercetin-3-O-rutinoside	0.25	0.75	99.99	0.80	0.9999	370
	Kaempferol	0.15	0.45	99.16	1.75	0.9997	370
	Myricetin	0.02	0.06	93.99	1.19	0.9998	370
	Quercetin	0.03	0.09	100.00	0.62	0.9997	370
	Quercetin-3-O-glucoside	0.06	0.18	100.01	0.25	0.9989	370
Anthocyanins	Cyanidin-3-O-glucoside	0.30	0.90	99.98	0.46	0.9999	530
	Cyanidin-3-O-rutinoside	0.60	1.80	99.89	1.22	0.9998	530
